# Long term follow-up in patients with initially diagnosed low grade Ta non-muscle invasive bladder tumors: tumor recurrence and worsening progression

**DOI:** 10.1186/1471-2490-14-5

**Published:** 2014-01-08

**Authors:** Hiroaki Kobayashi, Eiji Kikuchi, Shuji Mikami, Takahiro Maeda, Nobuyuki Tanaka, Akira Miyajima, Ken Nakagawa, Mototsugu Oya

**Affiliations:** 1 Department of Urology, Keio University School of Medicine, 35 Shinanomachi, Shinjuku-ku, Tokyo 160-8582, Japan; 2 Division of Diagnostic Pathology, Keio University School of Medicine, 35 Shinanomachi, Shinjuku-ku, Tokyo 160-8582, Japan

**Keywords:** Bladder cancer, Intravesical instillation, Recurrence, Progression

## Abstract

**Background:**

We evaluated the clinical outcome of low grade Ta bladder cancer followed-up for a long period using the 2004 WHO grading system.

**Methods:**

We retrospectively reviewed 190 patients with primary, low grade Ta bladder cancer. We defined worsening progression (WP) as confirmed high grade Ta, all T1 or Tis/concomitant CIS of bladder recurrence, upper urinary tract recurrence (UTR), or progression to equal to or more than T2. The associations between clinicopathological factors and tumor recurrence as well as WP pattern were analyzed. We also evaluated the late recurrence of 76 patients who were tumor-free for more than 5 years.

**Results:**

Tumor recurrence and WP occurred in 82 (43.2%) and 21 (11.1%) patients during follow-up (median follow-up: 101.5 months), respectively. WP to high grade Ta, all T1 or Tis/concomitant CIS was seen in 17 patients, and UTR and progression to equal to or more than T2 were seen in 2 and 2 patients, respectively. Multivariate analyses demonstrated that multiple tumor (p < 0.001, HR: 2.97) and absence of intravesical instillation (IVI) (p < 0.001, HR: 2.88) were significant risk factors for tumor recurrence while multiple tumor was the only risk factor for WP (p = 0.001, HR: 5.26). After a 5-year tumor-free period, 9 patients experienced late recurrence in years 5 and 10 and were diagnosed at a follow-up cystoscopy, however, only 2 patients recurred beyond 10 years and were found by gross hematuria. There were no significant risk factors of late recurrence.

**Conclusions:**

Multiple tumor was a risk factor for both tumor recurrence and WP while IVI did not affect the occurrence of WP. Our results suggest that routine follow-up of patients with low grade Ta bladder cancer is needed up to 10 years from the initial diagnosis.

## Background

Approximately 50 % of newly diagnosed cases of bladder cancer are low grade, noninvasive and papillary tumors [1]. The standard treatment for non-muscle invasive bladder cancer (NMIBC) is transurethral resection of the bladder tumor (TUR-BT) with or without adjuvant intravesical instillation (IVI) of chemotherapy or Bacillus Calmette-Guerin (BCG) therapy [2]. The most important problems associated with NMIBC are that they have high rates of recurrence and risk of progression. Approximately 50% to 70% of NMIBC have a recurrence within 5 years, and 5% to 20% progress to invasive tumors [3]. To the best of our knowledge, there have been no reports with longer follow-up data focusing on the effects of IVI on tumor recurrence and progression in patients with low grade Ta tumors based on the 2004 WHO classification [4,5]. Also, there are few published studies in which a large number of patients were followed for more than 5 years after the initial diagnosis. Only two papers provide information on the risk of recurrence and progression after a long tumor-free period [6,7]. Although some long follow-up studies also showed most tumors recurred or progressed within 5 years, recent data support the need for long term follow-up for more than 10–15 years in such patients even after an initial response to BCG therapy and a recurrence-free period for more than 5 years [6].

In this study, we analyzed the clinical outcome of initially diagnosed low grade Ta tumors after re-assessing all pathological specimens according to the 2004 WHO classification, with a special focus on tumor recurrence and worsening progression (WP) pattern and discuss the need for longer follow-up.

## Methods

We reviewed the medical records of 242 patients (male: 199, female: 43) who underwent TUR-BT with complete tumor resection for the past 30 years at Keio University Hospital for an initially diagnosed TaG1-2 tumor. We excluded 44 patients who had already undergone TUR-BT at another hospital or who had a history of upper tract urothelial cancer. After re-evaluation of all pathological specimens by a dedicated uro-pathologist, with a special focus on the 2004 WHO grading system, 8 patients with G2 tumors were re-classified as high grade. The remaining 190 NMIBC patients (male: 156, female: 34) who were initially diagnosed with a tumor that was low grade Ta were included in the current analysis. All of the tumors were histologically confirmed as urothelial carcinoma.

These patients were followed by urine cytology and cystoscopy at 3-month intervals during the initial year, every 6 months for the next 5 years, and then yearly thereafter. Intravenous urography, ultrasonography, and/or CT scanning were used to evaluate distant metastasis and upper urinary tract recurrence (UTR) every 1 or 2 years for 5 years. Recurrence was defined as the occurrence of a new tumor in the bladder. Worsening progression (WP) was defined as confirmed (1) high grade Ta, all T1, or Tis/concomitant CIS of bladder recurrence, (2) UTR, or (3) progression to equal to or more than T2.

The use of adjuvant therapies including intravesical BCG or mitomycin (MMC) instillation depended primarily on the discretion of the attending physician. In the overall patient population, BCG and MMC therapies were performed in 71 patients (37.4%) and 12 patients (6.3%), respectively. This study was approved by Keio university hospitals ethical committee. We obtained the patient’s informed consent including their approval for potential use of their anonymized medical data from our data base for research and audit purposes.

The following were analyzed for each individual patient: age, gender, multiplicity and smoking status. Smoking status was classified as 1) nonsmokers; those who had never smoked during their lifetime, 2) ex-smokers; those who had quit smoking before the diagnosis and 3) current smokers; those who still smoked regularly at the initial TUR-BT. Recurrence-free survival rate curves were constructed using the Kaplan-Meier method, and were compared using the log-rank test. Differences among groups were regarded as significant when p < 0.05. Univariate and multivariate analyses of data were performed using the Cox proportional hazards model with stepwise forward selection. These analyses were performed with a statistical software package (SPSS, version 19.0).

## Results

### Tumor recurrence and worsening progression rate in entire patient population

**Figure 1:**
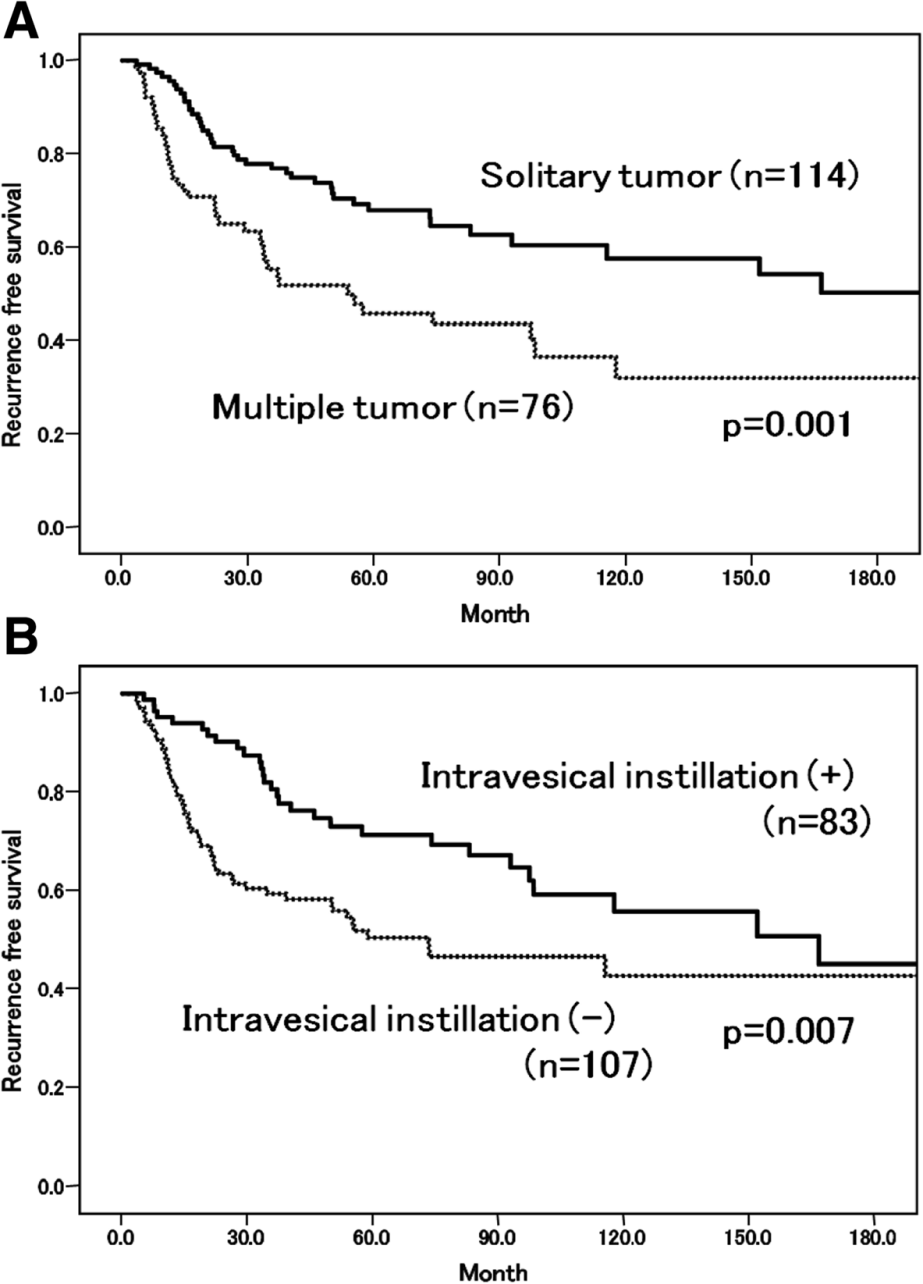
Recurrence-free survival rates (A) by tumor multiplicity, (B) by presense of adjuvant intravesical instillation.

The mean age of the patients was 62.9 years (range, 22–89) and the median follow-up interval was 101.5 months (range, 11.1 to 298.2). Solitary/multiple tumors were seen in 114/76 patients, respectively. Tumor recurrence occurred in 82 patients (43.2%). Most patients who had tumor recurrence could be diagnosed by the routine follow-up cystoscopic examination except for 3 patients (3.7%) who were detected due to gross hematuria. When we divided the patients into two groups, those with or without tumor recurrence, there were no significant differences in age, gender, IVI or smoking status between the two groups (Table 1). The recurrence rate in multiple tumors (53.9%) was significantly higher than that in solitary tumors (36.0%). Univariate and multivariate analyses demonstrated that multiple tumor and absence of IVI were significant risk factors for tumor recurrence (Table 2). Kaplan-Meier curves demonstrated that the 5-year recurrence free survival rate for solitary tumors (68.0%) was significantly higher than that for multiple tumors (45.9%, p = 0.001) (Figure 1A), and also higher for patients receiving intravesical instillation (71.3% vs. 50.3% without IVI, p = 0.007) (Figure 1B).

**Table 1:** Clinical characteristics of all 190 patients

		**Total**	**Recurrence (+)**	**Recurrence (−)**	***p *** **value**	**WP (+)**	**WP (−)**	***p *** **value**
N		190	82	108		21	169	
Age (y)	Mean Range	62.9	63.0	62.9		64.9	62.7	
		22-89	26-83	22-89		47-83	22-89	
	≤70	128	56	72	NS	14	114	NS
	>70	62	26	36		7	55	
Gender	Male	156	72	84	NS	19	137	NS
	Female	34	10	24		2	32	
Multiplicity	Solitary	114	41	73	0.017	7	107	0.016
	Multiple	76	41	35		14	62	
IVI	Yes	83	29	54	NS	11	72	NS
	No	107	53	54		10	97	
Smoking	None	100	40	60	NS	10	90	NS
	Current	56	29	27		9	47	
	Ex-smoker	27	10	17		2	25	
	Unknown	7	3	4		0	7	

*IVI* intravesical instillation, *WP* worsening progression, *NS* non-significant.

**Table 2:** Univariate and multivariate analyses for tumor recurrence and WP in overall patient population

	**Recurrence**			**WP**		
	**Univariate**	**Multivariate**		**Univariate**	**Multivariate**	
	**p value**	**p value**	**HR**	**p value**	**p value**	**HR**
Age (≤70 vs. >70)	0.465			0.444		
Gender (male vs. female)	0.152			0.412		
Multiplicity (solitary vs. multiple)	0.001	<0.001	2.97	0.003	0.001	5.26
Treatment (IVI vs. observation)	0.007	<0.001	2.88	0.454		
Smoking (nonsmoker vs. smoker)	0.501			0.606		

*IVI* intravesical instillation, *WP* worsening progression, *smoker*: current smoker + ex-smoker.

Overall WP occurred in 21 patients (11.1%) and the average time to WP was 82.4 months (range, 5.6-298.2). WP to high grade Ta, all T1, or Tis/concomitant CIS of bladder recurrence was seen in 4, 8 and 5 patients, respectively, UTR was seen in 2 patients (1.1%), and progression to equal to or more than T2 was observed in 2 patients (1.1%). Also, there were various types of timing and intervals for WP. Twenty of 21 patients (95.2%) experienced WP until the 2^nd^ recurrence and 12 patients (57.1%) experienced WP on the 1^st^ recurrence. All 8 patients who experienced WP on the 2^nd^ recurrence had a low grade Ta type tumor on the 1^st^ recurrence, and the average time between the 1^st^ and 2^nd^ recurrence was 22.9 months. An exception was one patient who experienced minor recurrences three times and WP occurred on the 4^th^ recurrence. The three recurrences were all low grade Ta, and in the end WP to high grade T1 occurred although intravesical chemotherapy and BCG instillations were performed after every TUR-BT.

**Figure 2:**
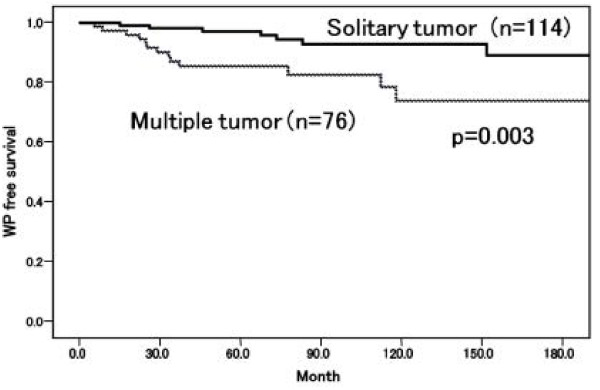
WP-free survival rates by tumor multiplicity.

When we divided the patients into two groups, those with or without WP, there were no significant differences in age, gender, IVI or smoking status between the two groups (Table 1). WP occurred more often in the multiple tumor group (18.4%) than in the solitary tumor group (6.1%) and the difference was significant (p = 0.016). Univariate and multivariate analyses demonstrated that multiple tumor was the only significant risk factor for WP (Table 2). Kaplan-Meier curve demonstrated that the 5-year WP free survival rate for solitary tumors (97.2%) was significantly higher than that for multiple tumors (85.5%, p = 0.003) (Figure 2).

### Late recurrence beyond 5-year tumor-free period

We next focused on patients who were tumor-free for more than 5 years from initial TUR-BT to first tumor recurrence and WP, called “late recurrence” and “late WP”. We identified 76 patients in this category, among whom adjuvant IVI had been performed in 40 (52.6%) patients (Table 3). The mean age of the patients was 61.1 years (range, 22–84), and solitary/multiple tumors were seen in 53/23 patients, respectively. Eleven patients (14.5%) experienced late recurrence, and of them, 5 patients (6.6%) had late WP (Table 4). There were no significant differences in age, gender, multiplicity, smoking status or adjuvant IVI performed in patients with or without late recurrence, and there were no significant risk factors of late recurrence. The average time to late recurrence and late WP was 103.5 and 104.5 months, respectively. Nine of 11 patients whose cancer recurred in years 5 and 10 in our study were diagnosed at a follow-up cystoscopy. Meanwhile, recurrence in 2 patients who were tumor-free beyond 10 years was found by gross hematuria. WP to high grade Ta, low grade T1, Tis, UTR, and high grade T3 were observed in one case in each. Also, 4 patients experienced WP on the first late recurrence while only one patient did on the 2^nd^ recurrence.

**Table 3:** Clinical characteristics of 76 patients with a tumor-free period for more than 5 years from initial diagnosis

		**Total**	**Late recurrence (+)**	**Late recurrence (−)**	***p *** **value**
N		76	11	65	
Age (y)	Mean	61.1	64.1	60.6	
	Range	(21.8-84.2)	(56.9-78.1)	(21.8-84.2)	
	≤70	58	9	49	NS
	>70	18	2	16	
Gender	Male	60	10	50	NS
	Female	16	1	15	
Multiplicity	Solitary	53	7	46	NS
	Multiple	23	4	19	
IVI	Yes	40	8	32	NS
	No	36	3	33	
Smoking	None	44	6	38	NS
	Current	23	3	20	
	Ex-smoker	8	1	7	
	Unknown	1	1	0	

**Table 4:** Clinical data for patients with late recurrence and late WP

**Patient no.**	**Age (yr)**	**Gender**	**Smoking status**	**Multiplicity**	**Tumor-free period (mo)**	**Pathology on late rec./WP**	**Follow up (mo)**	**Status of last contact**
1	57	Male	Current	M	98.4	S, low, Ta	232.6	Alive, NED
2	58	Male	None	M	117.8	M, high, Tis	172.1	Alive, cystectomy performed
3	58	Male	Ex-smoker	M	74.0	S, low, Ta	298.2	Alive, NED
4	59	Female	None	M	97.4	M, low, Ta	118.2	Alive, UTR, NUx performed
5	60	Male	None	S	166.6	S, low, Ta	178.4	Alive, NED
6	61	Male	None	S	73.5	S, low, Ta	273.6	Alive, NED
7	62	Male	Current	S	73.3	S, high, Ta	244.3	Alive, NED
8	67	Male	Unknown	S	92.9	M, low, Ta	134.8	Alive, NED
9	68	Male	Current	S	151.8	M, low, T1	206.4	Alive, NED
10	76	Male	None	S	115.5	S, low, Ta	176.0	Alive, NED
11	78	Male	None	S	83.1	M, high, T3	96.8	Dead of intercurrent disease

*M* multiple, *S* solitary, *WP* worsening progression, *NED* no evidence of disease, *UTR* upper urinary tract recurrence, *NUx* nephroureterectomy.

## Discussion

We reviewed 190 patients with primary, low grade Ta NMIBC patients and evaluated whether patient-related factors (age, gender, multiplicity, smoking status and adjuvant treatment) were associated with tumor recurrence and WP. Multivariate analysis demonstrated that multiplicity was a risk factor for both tumor recurrence and WP, and that IVI did not affect the occurrence of WP. While none of the patients died of bladder cancer during follow-up, late recurrence and late WP occurred in 11 and 5 patients, respectively.

Zieger et al. presented the natural history of 212 patients initially diagnosed with TaG1-2 tumors for up to 20 years. Only 14 patients received intravesical instillation in their study. Ten of the 212 (4.7%) developed into TaG3 or CIS, 18 (8.5%) developed into T1, and 23 (10.8%) showed muscle invasion or distant metastases [8]. According to our definition of WP, WP was seen in 24.1% in their study, which was relatively high com-pared to our study. Similarly, Prout et al. followed 178 patients with TaG1 bladder tumors for up to 10 years. They reported that a change in grade or stage progression occurred in 13 (7.3%) patients, while only 14 patients (7.9%) received intravesical chemotherapy [9]. Akagashi et al. reported no patients initially diagnosed with TaG1-2 tumors progressed to muscle invasive tumors, while 6 of 62 (9.7 %) patients developed into Tis or T1. One reason for this low percentage of progression was that most of the patients received intravesical chemotherapy for more than 2 years [10]. From these reports, the recurrence rate of initially diagnosed TaG1-2 bladder cancer was 50-60%, and the WP rate was highly variable (between 7% and 24%). In our population of initially diagnosed low grade Ta bladder tumors, the recurrence rate and WP rate were 43.2% and 11.1%, respectively.

We reviewed longer follow-up data for a maximum of 25 years and re-assessed all pathological specimens using the 2004 WHO classification. Only 8 of 198 (4.0%) of G1-2 tumors were re-classified as high grade in our study. Miyamoto et al. evaluated low grade papillary urothelial carcinoma after re-classifying all specimens and reported that 8 of 55 patients (14.5%) were re-classified as having high grade tumors [11]. Pellucchi et al. evaluated tumor recurrence and progression with both the 1973 and 2004 WHO grading systems in patients with primary low grade Ta NMIBC and concluded that the 1973 WHO grading system predicted the risk of recurrence more accurately than the 2004 system and the 2 classifications showed the same accuracy for predicting the risk of progression [12]. The 2013 EAU guideline states that both grading classifications should be used until the 2004 WHO system is validated by more prospective trials [13].

Holmang et al. reported the outcomes in patients treated with BCG intravesical therapy who were tumor-free for more than 5 years (N = 204). Of the 204 patients, 110 (53.9%) had a G1 or G2 tumor. They stated that patients with TaG1-2 tumors treated with BCG have a very good long-term prognosis, but late recurrences were observed. Furthermore, as all low grade recurrences were diagnosed at a follow-up cystoscopy and office cystoscopy generally is a simple procedure, they concluded that continuing to follow patients with TaG1-2 for more than 5 years is encouraging [6]. Our results support their findings. All patients in our study who experienced recurrence in years 5 and 10 were diagnosed at a follow-up cystoscopy. Meanwhile, recurrence in 2 patients who had been tumor-free beyond 10 years was found by gross hematuria. These results suggest that follow-up cystoscopy can be discontinued around 10 years from the initial diagnosis in patients with low grade Ta bladder cancer.

Smoking status is a well-known risk factor for poor outcome in bladder cancer and the strong association between smoking and primary NMIBC recurrence was observed in previous studies [14-16]. However, our results revealed that smoking status is not associated with bladder recurrence rate, WP rate, or late recurrence rate. One of the reasons for our negative result is that the relatively lower percentage of smokers and lower amount of smoking in Japanese NMIBC populations. Further studies with a larger population are warranted in order to evaluate the association between smoking status and tumor outcome in low grade Ta NMIBC.

The present study has several limitations. First, it was performed in a retrospective manner with a limited number of patients, thus unknown sources of bias may exist in the findings. However, since we re-reviewed all pathological specimens and reclassified them as absolute low grade tumors, our results represent more reliable data compared to data obtained before re-evaluation. Second, in our database, tumor size/volume was not included routinely because of the inaccuracy of measurements of tumor size by cystoscopic findings. Finally, we did not provide all patients with a single immediate postoperative instillation of chemotherapy within 24 h or any maintenance intravesical therapies, which may have improved the results.

## Conclusions

The tumor recurrence rate and WP rate in patients with primary, low grade Ta bladder cancer were 43.2 % and 11.1 %, respectively. Multiple tumor was a risk factor for both tumor recurrence and WP, while IVI did not affect the occurrence of WP. The late recurrence rate and late WP rate were 14.5 % and 6.6 %, respectively. Our results suggest that routine follow-up of patients with low grade Ta bladder cancer is needed up to 10 years from the initial diagnosis.

## Competing interests

The authors declare that they have no competing interests.

## Authors’ contributions

HK, TM and NT formulated database. HK performed the initial analyses and drafted the first manuscript. All authors assisted in the analysis and interpretation of data. EK conceived of the study, and participated in its design and coordination and helped to draft the manuscript. All authors read and approved the final manuscript.

## Pre-publication history

The pre-publication history for this paper can be accessed here:

http://www.biomedcentral.com/1471-2490/14/5/prepub

## References

[B1] Greenlee RT Murray T Bolden S Wingo PA Cancer statistics, 2000 CA Cancer J Clin 2000 50 1 7 33 10.3322/canjclin.50.1.7 10735013

[B2] Hendricksen K Witjes JA Current strategies for first and second line intravesical therapy for nonmuscle invasive bladder cancer Curr Opin Urol 2007 17 5 352 357 10.1097/MOU.0b013e3281c55f2b 17762630

[B3] Donat SM Evaluation and follow-up strategies for superficial bladder cancer Urol Clin North Am 2003 30 4 765 776 10.1016/S0094-0143(03)00060-0 14680313

[B4] MacLennan GT Kirkali Z Cheng L Histologic grading of noninvasive papillary urothelial neoplasms Eur Urol 2007 51 4 889 897 10.1016/j.eururo.2006.10.037 17095142

[B5] Epstein JI Amin MB Reuter VR Mostofi FK The World Health Organization/International Society of Urological Pathology consensus classification of urothelial (transitional cell) neoplasms of the urinary bladder. Bladder Consensus Conference Committee Am J Surg Pathol 1998 22 12 1435 1448 10.1097/00000478-199812000-00001 9850170

[B6] Holmang S Strock V Should Follow-up Cystoscopy in Bacillus Calmette-Guerin-Treated Patients Continue After Five Tumour-Free Years? Eur Urol 2012 61 3 503 507 10.1016/j.eururo.2011.11.011 22119022

[B7] Matsumoto K Kikuchi E Horiguchi Y Tanaka N Miyajima A Nakagawa K Nakashima J Oya M Late recurrence and progression in non-muscle-invasive bladder cancers after 5-year tumor-free periods Urology 2010 75 6 1385 1390 10.1016/j.urology.2009.09.088 20110108

[B8] Zieger K Wolf H Olsen PR Hojgaard K Long-term follow-up of noninvasive bladder tumours (stage Ta): recurrence and progression BJU Int 2000 85 7 824 828 10792160 10.1046/j.1464-410x.2000.00547.x

[B9] Prout GR Jr Barton BA Griffin PP Friedell GH Treated history of noninvasive grade 1 transitional cell carcinoma. The National Bladder Cancer Group J Urol 1992 148 5 1413 1419 1433540 10.1016/s0022-5347(17)36924-0

[B10] Akagashi K Tanda H Kato S Ohnishi S Nakajima H Nanbu A Nitta T Koroku M Sato Y Hanzawa T Recurrence pattern for superficial bladder cancer Int J Urol 2006 13 6 686 691 10.1111/j.1442-2042.2006.01386.x 16834643

[B11] Miyamoto H Brimo F Schultz L Ye H Miller JS Fajardo DA Lee TK Epstein JI Netto GJ Low-grade papillary urothelial carcinoma of the urinary bladder: a clinicopathologic analysis of a post-World Health Organization/International Society of Urological Pathology classification cohort from a single academic center Arch Pathol Lab Med 2010 134 8 1160 1163 20670136 10.5858/2009-0403-OA.1

[B12] Pellucchi F Freschi M Ibrahim B Rocchini L Maccagnano C Briganti A Rigatti P Montorsi F Colombo R Clinical reliability of the 2004 WHO histological classification system compared with the 1973 WHO system for Ta primary bladder tumors J Urol 2011 186 6 2194 2199 10.1016/j.juro.2011.07.070 22019037

[B13] Babjuk M Burger M Zigeuner R Shariat SF van Rhijn BW Comperat E Sylvester RJ Kaasinen E Bohle A Palou Redorta J EAU guidelines on non-muscle-invasive urothelial carcinoma of the bladder: update 2013 Eur Urol 2013 64 4 639 653 10.1016/j.eururo.2013.06.003 23827737

[B14] Fleshner N Garland J Moadel A Herr H Ostroff J Trambert R O'Sullivan M Russo P Influence of smoking status on the disease-related outcomes of patients with tobacco-associated superficial transitional cell carcinoma of the bladder Cancer 1999 86 11 2337 2345 10.1002/(SICI)1097-0142(19991201)86:11<2337::AID-CNCR23>3.0.CO;2-6 10590376

[B15] Lammers RJ Witjes WP Hendricksen K Caris CT Janzing-Pastors MH Witjes JA Smoking status is a risk factor for recurrence after transurethral resection of non-muscle-invasive bladder cancer Eur Urol 2011 60 4 713 720 10.1016/j.eururo.2011.07.010 21794974

[B16] Rink M Furberg H Zabor EC Xylinas E Babjuk M Pycha A Lotan Y Karakiewicz PI Novara G Robinson BD Impact of smoking and smoking cessation on oncologic outcomes in primary non-muscle-invasive bladder cancer Eur Urol 2013 63 4 724 732 10.1016/j.eururo.2012.08.025 22925575 PMC3969986

